# Deep learning approach for analyzing chest x-rays to predict cardiac events in heart failure

**DOI:** 10.3389/fcvm.2023.1081628

**Published:** 2023-05-19

**Authors:** Kenya Kusunose, Yukina Hirata, Natsumi Yamaguchi, Yoshitaka Kosaka, Takumasa Tsuji, Jun’ichi Kotoku, Masataka Sata

**Affiliations:** ^1^ Department of Cardiovascular Medicine, Tokushima University Hospital, Tokushima, Japan; ^2^ Ultrasound Examination Center, Tokushima University Hospital, Tokushima, Japan; ^3^ Department of Radiological Technology, Graduate School of Medical Care and Technology, Teikyo University, Tokyo, Japan

**Keywords:** heart failure with reduced ejection fraction, heart failure with preserved ejection fraction, artificial intelligence, deep learning, chest x-ray

## Abstract

**Background:**

A deep learning (DL) model based on a chest x-ray was reported to predict elevated pulmonary artery wedge pressure (PAWP) as heart failure (HF).

**Objectives:**

The aim of this study was to (1) investigate the role of probability of elevated PAWP for the prediction of clinical outcomes in association with other parameters, and (2) to evaluate whether probability of elevated PAWP based on DL added prognostic information to other conventional clinical prognostic factors in HF.

**Methods:**

We evaluated 192 patients hospitalized with HF. We used a previously developed AI model to predict HF and calculated probability of elevated PAWP. Readmission following HF and cardiac mortality were the primary endpoints.

**Results:**

Probability of elevated PAWP was associated with diastolic function by echocardiography. During a median follow-up period of 58 months, 57 individuals either died or were readmitted. Probability of elevated PAWP appeared to be associated with worse clinical outcomes. After adjustment for readmission score and laboratory data in a Cox proportional-hazards model, probability of elevated PAWP at pre-discharge was associated with event free survival, independent of elevated left atrial pressure (LAP) based on echocardiographic guidelines (*p* < 0.001). In sequential Cox models, a model based on clinical data was improved by elevated LAP (*p *= 0.005), and increased further by probability of elevated PAWP (*p *< 0.001). In contrast, the addition of pulmonary congestion interpreted by a doctor did not statistically improve the ability of a model containing clinical variables (compared *p *= 0.086).

**Conclusions:**

This study showed the potential of using a DL model on a chest x-ray to predict PAWP and its ability to add prognostic information to other conventional clinical prognostic factors in HF. The results may help to enhance the accuracy of prediction models used to evaluate the risk of clinical outcomes in HF, potentially resulting in more informed clinical decision-making and better care for patients.

## Introduction

Heart failure (HF) continues to be a significant socioeconomic issue and is one of the top causes of death from cardiovascular disease (CV) (1). Despite the development of current therapy, readmission rates for HF have remained high (2). The identification of hospitalized patients with a high risk of HF readmission is important for providing timely interventions. Understanding the underlying etiology, severity, and prognosis of HF requires evaluation of CV imaging (3, 4). A standard chest x-ray (CXR) in patients with suspected HF has a certain clinical value in the diagnosis and management of HF (5, 6). However, the sensitivity and specificity of this imaging modality is relatively low (7, 8).

Recently, artificial intelligence (AI) including deep learning (DL) has been used to provide precise recognition of understated patterns in medical images (9, 10). We reported that a DL model based on CXR analysis predicted elevated pulmonary artery wedge pressure (PAWP) in patients who had undergone right heart catheterization (11). The probability of elevated PAWP may therefore be a potential tool for managing HF in the clinical setting. We hypothesize that a previously developed application of a CXR-based DL algorithm could also be used to predict re-hospitalized HF in patients with HF. The aims of the current study were (1) to investigate the potential of probability of elevated PAWP for the prediction of clinical outcomes in association with other parameters, and (2) to evaluate whether probability of elevated PAWP based on AI added prognostic information to other clinical prognostic factors in patients with HF.

## Methods

### Study population

**Figure 1:**
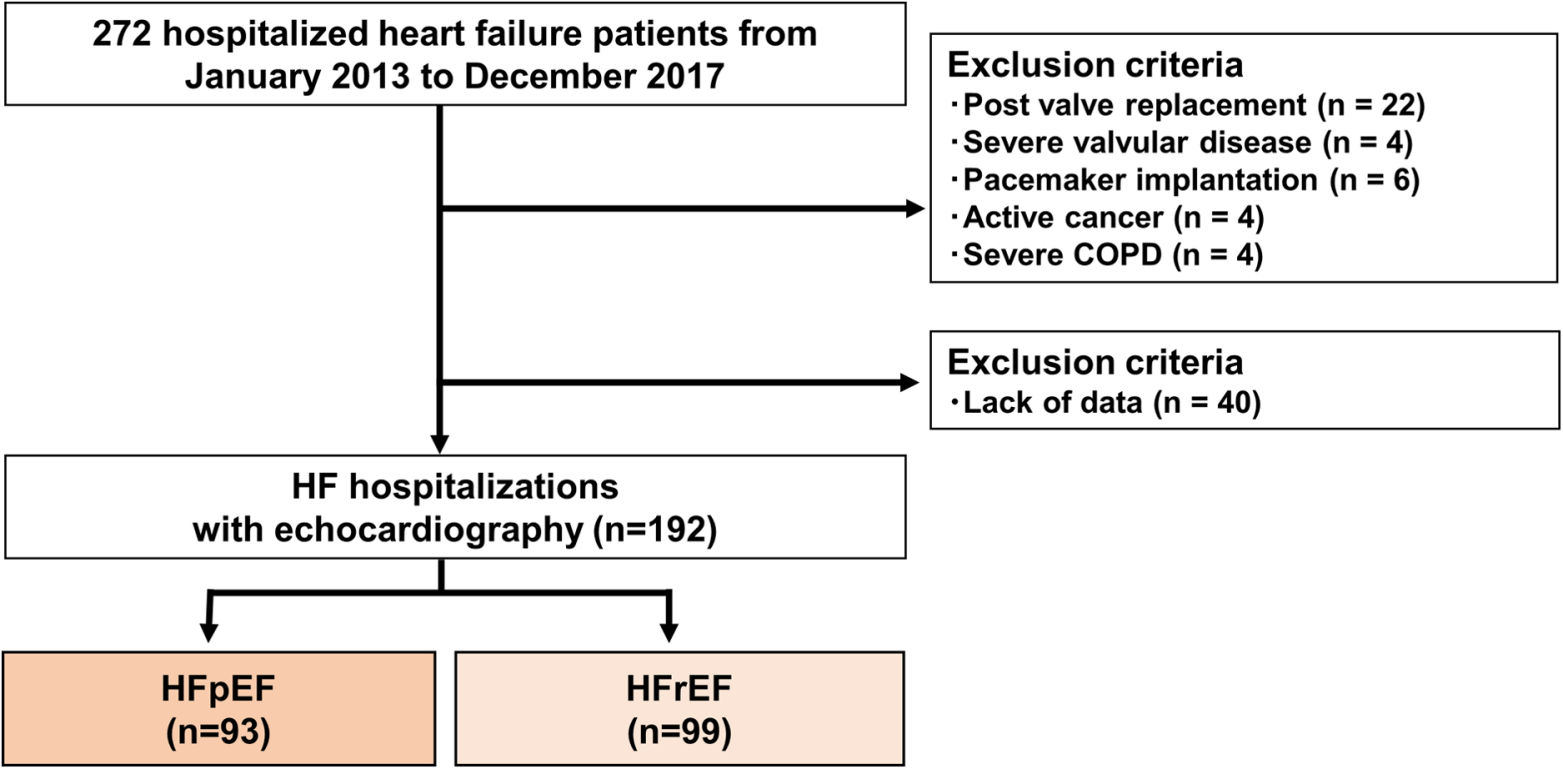
Flow chart showing recruitment of the patients. COPD, chronic obstructive pulmonary disease; HFpEF, heart failure with preserved ejection fraction; HFrEF, heart failure with reduced ejection fraction.

A single-center, retrospective study was designed (Figure 1). Two hundred seventy-two patients who were first HF hospitalized were enrolled initially. The study's time frame was from January 2013 to December 2017. Patients with HF were defined as having a clear history of HF with typical symptoms that were accompanied by signs including pulmonary congestion and BNP elevation (12). Exclusion criteria were post valve replacement, pacemaker implantation, active cancer, severe valvular disease and severe chronic obstructive pulmonary disease. Patients without clinical data at discharge were excluded. After these exclusions, 192 HF hospitalized patients were included in the final analysis. We divided this cohort into two groups: HF with reduced ejection fraction (HFrEF, *n* = 99) and HF with preserved EF (HFpEF, *n* = 93). Left ventricular ejection fraction (LVEF) less than 50% was designated as HFrEF, whereas LVEF greater than 50% was designated as HFpEF (13, 14). Patients collected to build the AI model were not included in this study.

### Chest x-ray

The Radiology Department performed all chest radiographs. One attending cardiologist who had no prior knowledge of the patients' clinical information or hemodynamic status assessed the CXR images. A typical posteroanterior chest radiograph was used to measure the cardiothoracic ratio (CTR), which measures the size of the cardiac chambers. Consensus of two expert agreement of lung congestions on CXR images was used.

### AI model for detection of PH

We used a previously developed AI model based on CXR analysis to predict elevated PAWP and then the continuous output of a classification network as a probability of elevated PAWP in the study cohort (11). The study involved examining CXR data at admission and discharge. All patients underwent CXR within 24 h of admission and 48 h before discharge. The AUC of ResNet 50 for predicting elevated PAWP (mean PAWP >18 mmHg) was 0.77 in the study cohort (11). The batch size was set at 16, with the Adam optimizer used for training (15). The whole learning process was calculated by a graphics processing unit (Geforce RTX 2080 Ti 11 Gb, NVIDIA) using Ubuntu 18.04 and Chainer version 5.1.0. We performed gradient-weighted class activation mapping (Grad-CAM) to visualize how our model detected a PAWP >18 mmHg from a CXR of each patient (16).

### Echocardiographic assessment

Echocardiography was performed using commercially available ultrasound machines. The echocardiographic data were obtained during the hospitalization according to the recommendations of the American Society of Echocardiography (17). Apical two- and four-chamber images were included. The biplane method of disks in two dimensions was used to calculate the volumes of the left atrium (LA) and LV. The LA volume index (LAVi) and LVEF were determined using these volumes. Based on 2016 recommendations, we implemented a decision tree using the mean *E*/*e*′ ratio, tricuspid regurgitant: TR velocity, and LAVi to identify the existence of elevated LA pressure (LAP) (18). Three criteria are required to decide if there is raised LAP: *E*/*e*′ ratio >14, LAVI > 34 ml/m², TR velocity >2.8 m/s.

### Calculation of readmission risk scores

The Yale–CORE HF application [developed by Yale New Haven Health Services Corporation/Center for Outcomes Research and Evaluation (YNHHSC/CORE)] was used to determine the readmission risk for each patient (19). Readmission risk was calculated using 20 variables per patient, including demographic and historical variables abstracted from the medical record, admission physical examination variables, and laboratory and clinical variables (age, sex, in-hospital cardiac arrest, history of diabetes, previous HF, coronary artery disease, previous percutaneous coronary intervention, aortic stenosis, stroke, chronic obstructive pulmonary disease, prior diagnosis of dementia, systolic blood pressure, heart rate, respiratory rate, plasma sodium, creatinine, and glucose levels, blood urea nitrogen level, hematocrit, and LVEF). The risk scores could be calculated without any missing data.

### Clinical outcomes

At Tokushima University Hospital, all patients received follow-up care, with clinical follow-up visits occurring at least every three months. After the follow-up echocardiography, the follow-up period began and terminated in May 2021. At Tokushima University Hospital or one of its affiliated hospitals, all the patients received follow-up care. There was no patient lost to follow-up. The AI data had little influence on clinical management. The primary endpoint was cardiac death or readmission due to HF using predetermined criteria. HF readmission was defined as admission for a primary diagnosis of HF and CV death as passing away from a CV cause, such as a myocardial infarction, a cerebrovascular accident, or sudden cardiac death. Based on previously published reports (20, 21), we mainly used variables measured at pre-discharge to assess the prognostic values in the study.

### Statistical analysis

Categorical data were expressed as an absolute number and percentages, whereas continuous data were expressed as mean standard deviation. Based on the likelihood of an elevated PAWP (>50%) being normal or abnormal, the patients were split into two groups. The Mann-Whitney U test or the unpaired Student's t test, as applicable, was used to compare continuous variables. Depending on the situation, the Fisher's exact test or the 2 test were used to compare categorical variables. The probability of elevated PAWP was used to divide the patients into two groups for Kaplan–Meier analysis, with survival compared using the log-rank test. A median value of Δprobability of elevated PAWP was used as the definition of improved probability of elevated PAWP. We used a Cox proportional-hazard model to determine the factors associated with survival. The variables selected were based on previous knowledge for the assessment of prognosis in patients with HF. To ascertain the incremental value of the probability of elevated PAWP over clinical data in relation to the main endpoint, sequential Cox models were built. The incremental prognostic value was defined as an increase in the global log-likelihood *χ*^2^ of the model that was statistically significant. The assumption of proportional hazards was assessed by plotting the scaled Schoenfeld residuals for each independent variable against time to determine whether these correlations were nonsignificant. Time-dependent receiver operating characteristic (ROC) curves were used to calculate the C-statistic analyzed by the R package survival ROC. The DeLong method was used to compare the C-statistic. All statistical analyses were performed using SPSS 21.0 (SPSS, Chicago, IL, USA), MedCalc 19.5.6 (Mariakerke, Belgium), and R 3.3.3 (R Foundation for Statistical Computing, Vienna, Austria). A *P* value < 0.05 was considered statistically significant.

## Results

### Clinical backgrounds

Table 1 shows the baseline characteristics of the patients at discharge. A total of 192 hospitalized patients with HF (mean age 69 ± 14 years; 61% male) were divided into two groups: those with HFrEF and those with HFpEF. The patients were treated with an ACEi/ACE (65%), β-blocker (79%), or diuretics (73%). No significant difference was observed between the two groups for age, blood pressure, and comorbidities except for ischemic cardiomyopathy. The patients with HFrEF included a higher number of males, a higher use of β-blockers, increased brain natriuretic peptide (BNP) levels, and a larger LV size. Interestingly, there was no difference in CXR profiles including CTR, lung congestion, probability of elevated PAWP on admission and pre-discharge between the two groups.

**Table 1:** Clinical characteristics.

	All (*n* = 192)	HFpEF (*n* = 93)	HFrEF (*n* = 99)	*p* value
Age (years)	69 ± 14	71 ± 14	68 ± 14	0.07
Male, *n* (%)	117 (61%)	46 (49%)	71 (72%)	0.002
BSA (m^2^)	1.62 ± 0.23	1.58 ± 0.22	1.66 ± 0.22	0.02
Heart rate (beats/min)	86 ± 20	82 ± 20	89 ± 20	0.01
Systolic BP (mmHg)	126 ± 24	129 ± 26	122 ± 23	0.05
Diastolic BP (mmHg)	74 ± 17	72 ± 18	75 ± 16	0.16
Readmission for HF, *n* (%)	57 (30%)	30 (32%)	27 (27%)	0.45
**Backgrounds**				
Hypertension, *n* (%)	131 (68%)	69 (74%)	62 (63%)	0.09
Diabetes, *n* (%)	80 (42%)	37 (40%)	43 (43%)	0.61
Chronic atrial fibrillation, *n* (%)	35 (18%)	20 (22%)	15 (15%)	0.26
Ischemic cardiomyopathy, *n* (%)	43 (22%)	11 (12%)	32 (32%)	<0.001
**Laboratory data**				
Hb (g/dl)	12.1 ± 2.3	11.9 ± 2.4	12.2 ± 2.2	0.25
eGFR (ml/min/1.73 m^2^)	50 ± 25	50 ± 26	50 ± 24	0.94
BNP (pg/ml)	228 (92, 471)	192 (62, 350)	291 (127, 531)	0.002
**Chest x-ray on pre-discharge**				
CTR	56 ± 7	55 ± 8	56 ± 7	0.17
Lung congestion, *n* (%)	53 (28%)	26 (26%)	27 (27%)	0.88
**Echocardiographic parameters**				
LVEF (%)	45 ± 15	59 ± 7	32 ± 7	–
LVEDVi (ml/m^2^)	83 ± 32	64 ± 26	101 ± 25	<0.001
LAVi (ml/m^2^)	51 ± 19	50 ± 19	52 ± 19	0.57
*E*/*e*′ ratio	13.8 ± 8.3	13.4 ± 8.2	14.2 ± 8.4	0.49
TR-V (m/s)	2.48 ± 0.46	2.55 ± 0.44	2.40 ± 0.47	0.02
Elevated LAP (%)	102 (53%)	50 (54%)	52 (53%)	0.86
**AI parameters**				
Probability of elevated PAWP on admission (%)	76 (23, 95)	70 (10, 95)	84 (26, 96)	0.16
Probability of elevated PAWP on pre-discharge (%)	11 (2, 62)	7 (2, 64)	13 (2, 55)	0.91
ΔProbability of elevated PAWP (%)	26 (2, 68)	12 (1, 61)	36 (3, 77)	0.17

Data are presented as number of patients (percentage), mean ± SD or median (interquartile range).

BSA, body surface area; BP, blood pressure; HF, heart failure; ACEi/ARB, angiotensin-converting-enzyme inhibitor/angiotensin II receptor blocker; HB, hemoglobin; eGFR, estimated glomerular filtration rate; BNP, brain natriuretic peptide; LVEF, left ventricular ejection fraction; LVEDVi, left ventricular end-diastolic volume index; LAVi, left atrial volume index; *E*, early diastolic transmitral flow velocity; *e*′, early diastolic mitral annular motion; TR-V, tricuspid regurgitant velocity; LAP, left atrial pressure.

The characteristics and echocardiographic parameters of the two groups with and without an abnormal elevated PAWP at pre-discharge are shown in Table 2. In this analysis, LAVi (*p *= 0.03), TR velocity (*p *= 0.01), and the presence of elevated LAP (*p *= 0.008) were associated with an abnormal probability of elevated PAWP. This result indicated probability of elevated PAWP was linked to left ventricular diastolic function. In patients with a normal probability of elevated PAWP on pre-discharge, the median probability of elevated PAWP on admission was 69%, while change in probability of elevated PAWP from admission to pre-discharge (Δprobability of elevated PAWP) was 53%. On the other hand, in patients with an abnormal probability of elevated PAWP on pre-discharge, the median probability of elevated PAWP on admission was high (94%). The status of lung congestion in patients with a higher probability of elevated PAWP may not have been reduced at pre-discharge. Based on expert CXR assessments, lung congestion is more frequent in patients with an abnormal probability of elevated PAWP.

**Table 2:** Clinical and echocardiographic parameters between normal and abnormal probability of elevated PAWP on pre-discharge.

x-ray group	Normal probability of elevated PAWP on pre-discharge	Abnormal probability of elevated PAWP on pre-discharge	*p* value
Number	134	58	
**AI parameters**			
Probability of elevated PAWP on pre-discharge (%)	3 (1, 13)	81 (65, 95)	–
Probability of elevated PAWP on admission (%)	69 (14, 92)	94 (67, 99)	<0.001
ΔProbability of elevated PAWP (%)	53 (6, 87)	9 (0, 44)	<0.001
**Characteristics**			
Age (years)	70 ± 14	69 ± 14	0.79
Male, %	38 (66)	82 (59)	0.39
Heart rate (beats/min)	86 ± 21	86 ± 19	0.89
Systolic BP (mmHg)	125 ± 22	128 ± 29	0.54
Yale-CORE HF score	22 ± 4	23 ± 4	0.26
**Medications**			
ACEi or ARB, *n* (%)	87 (65)	37 (64)	0.88
β-blocker, *n* (%)	107 (80)	45 (78)	0.73
Diuretics, *n* (%)	96 (72)	44 (76)	0.54
**Laboratory data**			
eGFR (ml/min/1.73 m^2^)	52 ± 26	46 ± 22	0.12
BNP (pg/ml)	236 (91, 471)	210 (107, 464)	0.55
**Chest x-ray on pre-discharge**			
CTR	55 ± 8	58 ± 7	0.02
Lung congestion, *n* (%)	25 (19)	28 (48)	0.001
**Echocardiographic parameters**			
LVEF (%)	45 ± 15	46 ± 16	0.76
LVEDVi (ml/m^2^)	84 ± 32	83 ± 30	0.84
LAVi (ml/m^2^)	49 ± 18	56 ± 21	0.03
*E*/*e*′ ratio	13.1 ± 7.4	15.3 ± 9.9	0.13
TR-V (m/s)	2.41 ± 0.41	2.61 ± 0.54	0.01
Elevated LAP (%)	63 (47%)	39 (67%)	0.008

See abbreviations as in Table 1.

### Cardiac mortality and readmission to HF

**Figure 2:**
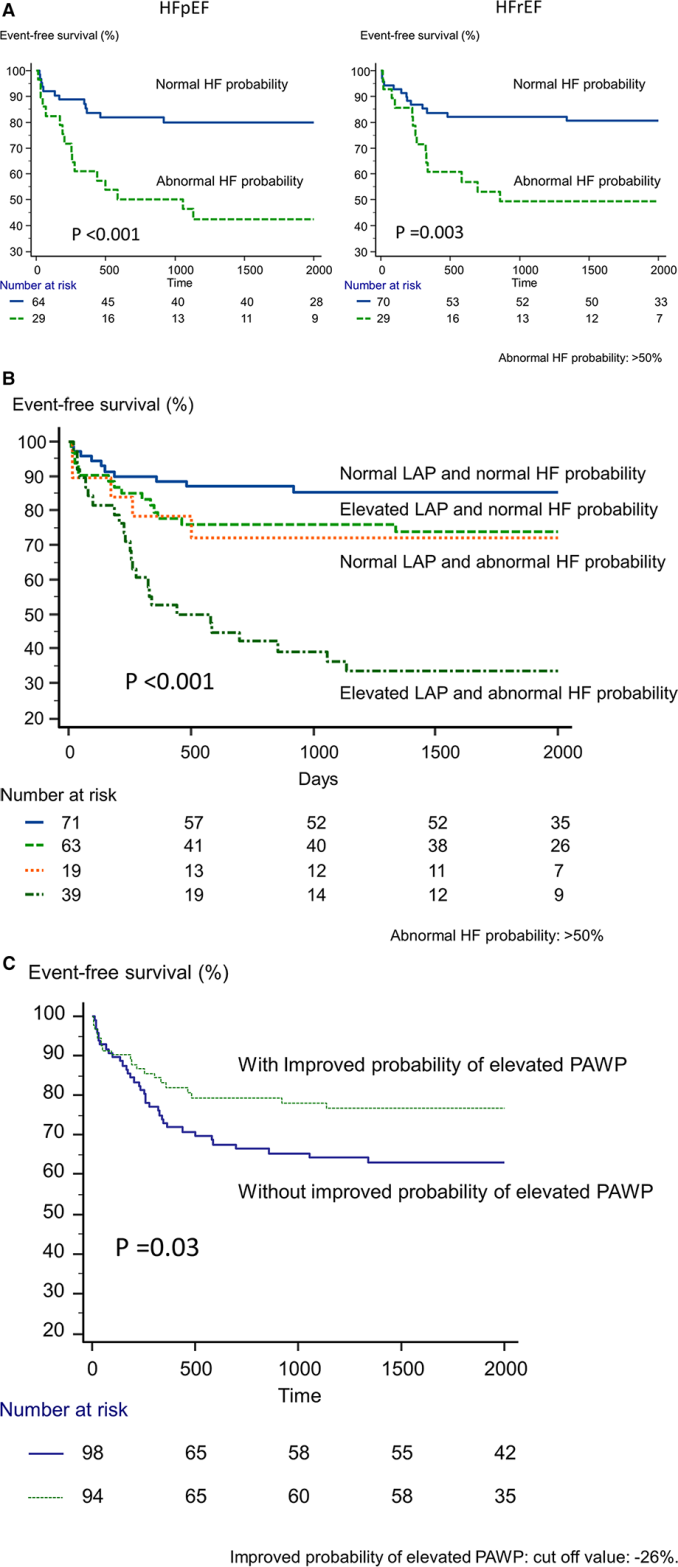
Kaplan-Meier analysis of event-free survival. (**A**) According to the presence or absence of abnormal probability of elevated PAWP in HFpEF and HFrEF, we divided patients into 2 groups. (**B**) According to the presence of an elevated left atrial pressure based on echocardiography and probability of elevated PAWP based on artificial intelligence, we divided patients into 4 groups. (**C**) According to the presence or absence of abnormal probability of elevated PAWP for improved and not-improved probability of elevated PAWP.

During a median follow-up period of 58 months (range, 11–80 months), 57 patients (30%) reached the primary endpoint (CV death, *n* = 13, or readmission due to HF, *n* = 44). During the follow-up period, no patient passed away from anything other than CV disease. Figure 2A shows the time to the primary endpoint. Probability of elevated PAWP appeared to be associated with worse clinical outcomes in both the HFpEF (*p* < 0.001) and HFrEF (*p *= 0.003) cohorts. Figure 2B shows the event-free survival of patients stratified according to the presence of an elevated LAP and abnormal probability of elevated PAWP (probability of elevated PAWP >50%). Patients with an elevated LAP and abnormal probability of elevated PAWP had significantly shorter event-free survival than those without these abnormalities (*p* < 0.001). In addition, Figure 2C shows the event-free survival of patients stratified according to improved or not improved probability of elevated PAWP (Δprobability of elevated PAWP, cut-off value: 26%). Patients without an improved probability of elevated PAWP had significantly shorter event-free survival than those with an improved probability of elevated PAWP (*p *= 0.03).

We used univariate and multivariate Cox proportional-hazard regression analysis to identify the variables connected to the main outcome. In the univariate model, the Yale-CPRE HF score, estimated glomerular filtration rate (eGFR), log BNP, LAVi, *E*/*e*′ ratio, TR-V, and elevated LAP as defined by the 2016 recommendations were linked to the primary endpoint (Table 3). The probability of elevated PAWP at admission was not related to the primary endpoint. Importantly, probability of elevated PAWP at pre-discharge (per 1SD) was related significantly with the primary outcomes (hazard ratio: 1.46, 95% CI: 1.23–1.72, *p* < 0.001). In addition, Δprobability of elevated PAWP from admission to pre-discharge was also associated with clinical outcomes. Pulmonary congestion by expert assessment was weakly associated with clinical outcomes (*p *= 0.049).

**Table 3:** Univariate associations of primary outcomes in hospitalized heart failure.

	HR (95%CI)	*p* value
**Characteristics**		
Age (years)	1.01 (0.99–1.03)	0.30
Male	0.97 (0.57–1.66)	0.92
Heart rate	0.99 (0.98–1.01)	0.25
Systolic BP	1.00 (0.99–1.01)	0.60
Yale-CORE HF score	1.08 (1.01–1.15)	0.03
**Medications**		
ACEi or ARB	0.86 (0.50–1.48)	0.59
β-blocker	0.80 (0.43–1.49)	0.48
Diuretics	1.55 (0.80–2.99)	0.19
**Laboratory data**		
eGFR (ml/min/1.73 m^2^)	0.98 (0.97–0.99)	0.003
Log BNP	2.66 (1.53–4.65)	0.001
**Chest x-ray on pre-discharge**		
CTR	0.37 (0.01–13.08)	0.58
Lung congestion	1.73 (1.00–2.98)	0.049
**Echocardiographic parameters**		
LVEF (%)	0.99 (0.98–1.01)	0.41
LVEDVi (ml/m^2^)	1.00 (0.99–1.01)	0.57
LAVi (ml/m^2^)	1.02 (1.00–1.03)	0.02
*E*/*e*′ ratio	1.04 (1.02–1.07)	0.001
TR-V (m/s)	1.85 (1.09–3.13)	0.02
Elevated LAP (%)	2.87 (1.59–5.18)	<0.001
**AI parameters**		
Probability of elevated PAWP on admission (per 1SD)	1.15 (0.94–1.41)	0.17
Probability of elevated PAWP on pre-discharge (per 1SD)	1.46 (1.23–1.72)	<0.001
ΔProbability of elevated PAWP (per 1SD)	0.71 (0.55–0.92)	0.01

HR, hazard ratio; CI, confidence interval; other abbreviations as in Table 1.

In the multivariate analysis (Table 4), both elevated LAP and probability of elevated PAWP based on the AI algorithm were significant predictors for the primary outcomes after adjustment for the Yale-CORE HF score, log BNP, and eGFR. Furthermore, the Δprobability of elevated PAWP was also a predictor for the primary endpoint after adjustment for these variables.

**Table 4:** Multivariate associations of primary outcomes in hospitalized heart failure.

	Model 1 (*χ*^2^: 24.4)			Model 2 (*χ*^2^: 31.4)			Model 3 (*χ*^2^: 41.1)		
	HR	95%CI	*p* value	HR	95%CI	*p* value	HR	95%CI	*p* value
**Clinical parameters**									
Yale-CORE HF score	1.01	0.93–1.09	0.86	1.02	0.94–1.10	0.70	1.01	0.93–1.09	0.86
Log BNP	1.90	1.03–3.52	0.04	1.99	1.07–3.70	0.03	1.97	1.11–3.50	0.02
eGFR	0.99	0.97–1.00	0.08	0.99	0.98–1.00	0.16	0.99	0.98–1.01	0.23
**Echocardiography**									
Elevated LAP	2.29	1.24–4.21	0.008	2.19	1.19–4.03	0.012	1.97	1.07–3.62	0.03
ΔProbability of elevated PAWP (per 1SD)				0.73	0.57–0.95	0.017			
Probability of elevated PAWP on pre-discharge (Per 1SD)							1.39	1.17–1.65	<0.001

HR, hazard ratio; CI, confidence interval; other abbreviations as in Table 1.

**Figure 3:**
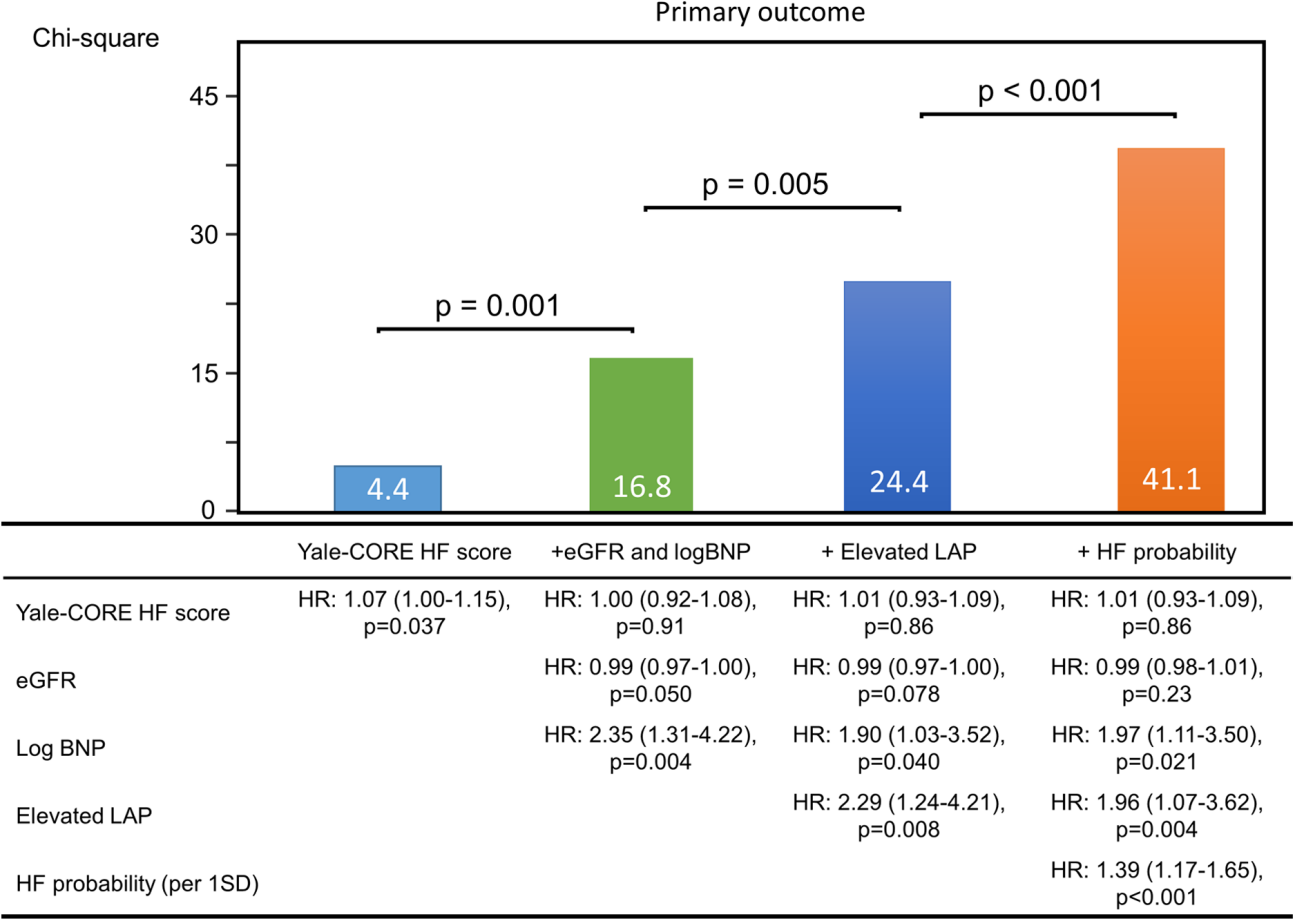
Incremental value of echocardiographic parameters. These figures illustrate the global *χ*^2^ of sequential Cox models that incorporated several clinical parameters. eGFR, estimate glomerular filtration rate; BNP, brain natriuretic peptide; LAP, left atrial pressure; HF, heart failure; HR, hazard ratio.

Figure 3 shows the added benefit of AI parameters for predicting the primary outcomes. The addition of echocardiographic assessment (elevated LAP) and probability of elevated PAWP significantly improved the ability of a model containing the Yale-CORE HF score, eGFR, and log BNP (model 1), Yale-CORE HF score, *χ*^2^ = 4.4 (model 2), plus eGFR and log BNP, *χ*^2^ = 16.8, *p *= 0.001 (model 3), plus elevated LAP, *χ*^2^ = 24.4, *p *= 0.005, plus probability of elevated PAWP on pre-discharge, *χ*^2^ = 41.1, *p *< 0.001). In contrast, the addition of pulmonary congestion interpreted by a doctor did not statistically improve the ability of a model containing the Yale-CORE HF score, eGFR, log BNP, and elevated LAP (model 3 plus pulmonary congestion, from *χ*^2^ = 24.4 to *χ*^2^ = 27.5, compared *p *= 0.086).

For the Cox model based on lung congestion by expert assessment, the Harrell C concordance statistic was calculated as 0.55 (95% CI: 0.49–0.61). The Harrell C concordance statistic was calculated as 0.72 (95% CI: 0.65–0.78) for the Cox model based on the Yale-CORE HF score, eGFR, pro BNP, and elevated LAP. When probability of elevated PAWP was added to the model, the C-statistic improved significantly to 0.78 (95% CI: 0.71–0.84, *p *= 0.039 for the comparison).

### Assessment of Grad-CAM

**Figure 4:**
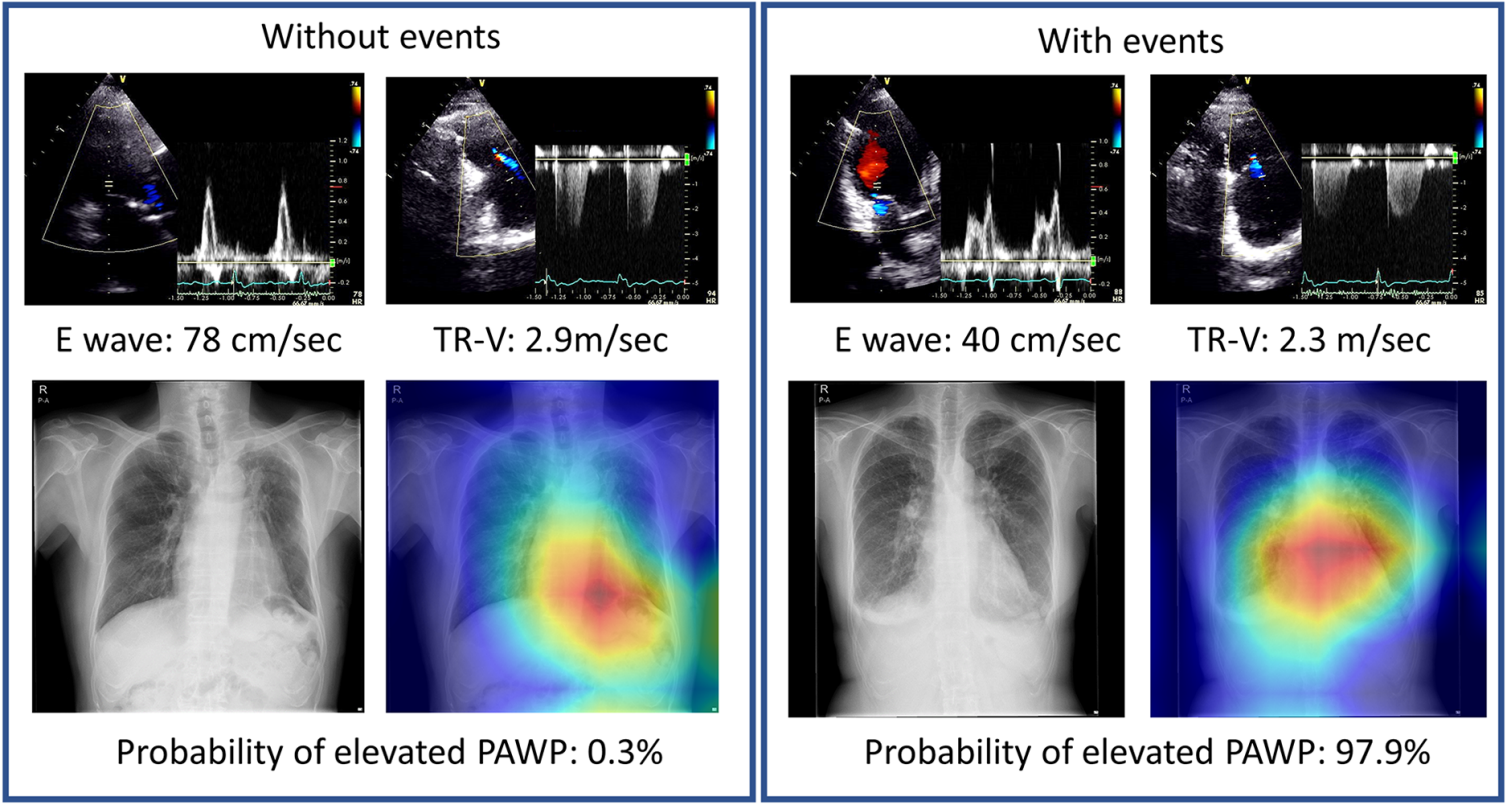
Representative cases with Grad-CAM images. Chest x-rays were visualized using Grad-CAM, with the yellow and red areas showing regions that the deep learning model considered important for probability of elevated PAWP.

We analyzed the images to determine where AI was focused to help explain the AI assessment (Figure 4). Grad-CAM demonstrated that in our situations, whether a patient had primary events or not, our model focused on the heart region. The proposed AI model may thus offer fresh perspectives to accurately identify differences in CXR images in the future large dataset.

## Discussion

The objective of this study was to assess the clinical meanings of probability of elevated PAWP based on an AI algorithm, as an association between probability of elevated PAWP and CV events. The study provided several insights into the interpretation of probability of elevated PAWP: (1) probability of elevated PAWP was related to left ventricular diastolic function; (2) patients with an abnormal probability of elevated PAWP had a significantly higher event rate compared to patients with a normal probability of elevated PAWP; (3) the association between probability of elevated PAWP and the primary endpoints remained significant after adjustment for HF risk score, laboratory data, and echocardiographic data. Interestingly, Lung congestion assessed by one attending cardiologist was only weakly associated with outcomes. This information might provide insights into the clinical utility of medical imaging based on an AI algorithm in patients with HF beyond assessments by experts. Our findings suggest that the likelihood of elevated PAWP may be helpful for clinical evaluation and follow-up during the ideal period of medical treatment.

### Findings on probability of elevated PAWP in chest x-rays

The association between classical radiographic features of HF in CXR images and physiological hemodynamic parameters has been described previously (22, 23). Cephalization of pulmonary venous blood flow occurs with redistribution of pulmonary blood flow and typically when the PAWP is >10–15 mmHg. Interstitial edema characterized by Kerley B lines is thought to result when the PAWP is >20 mmHg due to thickening of the interlobular septa. Alveolar edema is present when the PAWP exceeds 25 mmHg. However, these radiographic changes are not always present and sometimes may only be partially present, or indeed absent, even in cases of clinically significant HF. An increased cardiothoracic ratio is more common and more sensitive; however, it is less specific (24). Although these important findings may be present in CXR images, diagnostic limitations of the clinical and simple radiographic parameters are also observed in the clinical setting. In this study, the assessment of CXR by experts was not so strongly associated with outcomes.

Previously, we trained the AI model to detect an elevated PAWP >18 mmHg (11). Theoretically, an elevated probability of elevated PAWP based on AI can be associated with residual pulmonary congestion and cardiac enlargement. Based on our results, abnormal probability of elevated PAWP is associated with larger LA volumes, relatively higher *E*/*e*′ as a marker of LV filling pressure, higher tricuspid valve regurgitant velocity, and the proportion of elevated LAP (Table 2). Interestingly, the LV systolic function was not significantly associated with probability of elevated PAWP. Therefore, this index appears to be a sensitive marker of LV diastolic parameters in the clinical setting. Further studies are designed to clarify the detail of the hemodynamic mechanism for probability of elevated PAWP using simultaneous recordings of cardiac pressures measured using invasive catheters.

### Probability of elevated PAWP and outcomes

In univariate analysis, the Yale-CORE HF score, BNP level, renal function, and elevated LAP measured by echocardiography were associated with clinical events. The parameters are used to predict CV events, including HF rehospitalization. After adjustment for these known factors, the probability of elevated PAWP based on an AI algorithm was associated with the primary outcome. There is a possible explanation for the association between probability of elevated PAWP and worse clinical outcomes. Based on our results of congestive CXR images, probability of elevated PAWP appears to reflect elevated LA pressures. Several studies have shown that an elevated PAWP was associated significantly with CV events (25, 26). These associations possibly explain the association between probability of elevated PAWP and clinical events. More importantly, the changes in probability of elevated PAWP between admission and pre-discharge were also associated with clinical events. A recent publication from PARADIGM-HF showed that signs of persistent congestion observed in physical examinations provided significant independent prognostic value even beyond symptoms and the levels of natriuretic peptides (27). When patients who do not respond satisfactorily to HF therapy are confirmed by a pre-discharge CXR, further administration of diuretics or other intensive treatment for HF may be considered in the clinical setting. We found that probability of elevated PAWP at admission was not associated with subsequent clinical events and therefore concluded that pre-discharge assessment should be recommended for hospitalized HF patients in order to provide more information about their status.

### Artificial intelligence in the clinical setting

At present, many AI imaging studies estimate diagnostic accuracy using sensitivity and specificity (28), while there is limited data available to assess clinical outcomes. To help progress the study of AI in medical images it is necessary to assess the effects on clinically meaningful endpoints to improve applicability and allow effective deployment into clinical practice (29). In addition, it is essential to AI research to consistently use out-of-sample external validation and well-defined patient cohorts to augment the quality and interpretability of AI. In the present study we investigated an independent cohort with a previously published application of an AI model for probability of elevated PAWP used to provide prognostic value in patients with HF. We hope that AI imaging may be used in the near future not only for diagnostic accuracy but also for clinical utility (e.g., prediction of prognosis).

### Clinical utility of probability of elevated PAWP

The results of this study suggest that the probability of elevated PAWP based on AI algorithm provides incremental value to known parameters including clinical data, laboratory data and echocardiography. To our knowledge, this study is the first to examine the clinical efficacy of AI algorithms in HF patients and their relationship to cardiac events during follow-up. The probability of elevated PAWP will be significant in that it is simple, reproducible, measurable at almost all institutes and reflects prognostic power in heart failure. Model performance on prediction is significant at pre-discharge and poor at admission. The results are consistent with the finding that rehospitalization is less likely if congestion is well controlled (30). This model may play an important role as a guide for treatment of residual congestion in HF. Our data suggested that the changes in probability of elevated PAWP on x-rays during hospitalization may reflect the course of treatment for heart failure. We expect that it can be modeled and validated with multicenter data and used in clinical settings in the future.

### Limitations

The present study has several limitations. First, this was a single tertiary heart centers study with a small sample size in Japan. Therefore, the generalizability of the study findings was limited. On the other hand, we believed that the single-center study would have less biases than a study with a larger sample size, such as those caused by disparities in treatment effectiveness or a wide range of etiologies. Because there were so few events in the sample, there is a chance that the model will be overfit. Second, the cut-off value for abnormal probability of elevated PAWP (50%) was determined by our previous paper, thus, the accurate cut-off values may not be well organized in the different cohort. The validity and reliability of AI algorithms should improve in the near future with advances in machine learning and augmented data set. The study period was from 2013 to 2017. Some HF pharmacotherapies such as SGLT-2 inhibitors were not available routinely. Because this study was designed to evaluate the performance of AI for risk stratification of HF, it was not possible to assess this AI model in patients without HF. These limitations suggest that the current study should be considered as hypothesis-generating. Additional research is required to quantify the likelihood of elevated PAWP more fully in a multi-center large cohort that includes healthy populations.

## Conclusions

CXR assessment using the AI model may provide important incremental prognostic value for predicting readmission and cardiac mortality risk assessment in patients with HF compared with doctor-interpreted pulmonary congestion. The results may help to enhance the accuracy of prediction models used to evaluate the risk of clinical outcomes in HF, potentially resulting in more informed clinical decision-making and better care for patients.

## Data Availability

The raw data supporting the conclusions of this article will be made available by the authors, without undue reservation.
